# Synthesis, Biological Evaluation, and Modeling of Dimeric PPI Analogues as Novel DNA Minor Groove Binders

**DOI:** 10.3390/molecules13051179

**Published:** 2008-05-20

**Authors:** Yan-Hui Yang, Qing-He Wang, Han Nie, Hong Chen, Mao-Sheng Cheng

**Affiliations:** 1 Key Lab of New Drug Design and Discovery of Liaoning Province, School of Pharmaceutical Engineering, Shenyang Pharmaceutical University, Shenyang 110016, P. R. China; E-mails: yanhuiyang79@hotmail.com; qinghe-wang@163.com, nl13@sina.com; 2 Staff Room of Pharmacognosy, Medical College of Chinese People’s Armed Police Force, Tianjin 300162, P. R. China; E-mail: chenhongtian06@yahoo.com.cn

**Keywords:** Anti-tumor, Dimeric PPI analogues, DNA minor groove binders, Molecular modeling

## Abstract

A series of symmetrical dimeric proton pump inhibitor (PPI) analogues, designed as novel type DNA minor groove binders, was synthesized and evaluated for anti-tumor activity. Some of these new compounds showed IC_50_ values below 10 μM in an *in vitro* anti-tumor test. A molecular modeling study was performed to confirm the sequence selectivity of these compounds towards AT base pairs in DNA. Two effective compounds were selected and docked into the minor groove of DNA. The snug binding may be responsible for their cytotoxic and anti-tumor effects.

## Introduction

**Figure 1:**
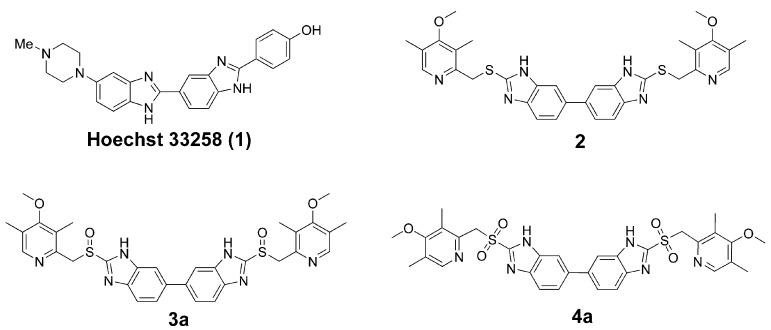
Lead compounds **1 – 2** and designed compounds **3a**, **4a**.

DNA minor groove binders (MGBs) are a novel family of anti-tumor agents and some of them have entered clinical trials. During the last decade, many synthetic minor groove binders have been reported, including analogues and conjugates of naturally occurring minor groove-binding agents, such as distamycin (Dst), netropsin (Net), CC-1065, anthramycin (Atm), and Hoechst 33258 [1]. Hoechst 33258 (**1**, Figure 1), a fluorescent reagent with a head-to-tail bis-benzimidazole structure, was initially found to be active against L1210 murine leukemia. During phase I trials in humans, some responses were seen in pancreatic cancer. However, a subsequent phase II trial did not show any objective responses [2]. X-ray crystallographic and NMR studies on complexes of Hoechst 33258 with AT-containing oligonucleotides have shown that the drug fits the minor groove snugly, with the planar benzimidazole groups oriented parallel to the direction of the groove and each inner-facing nitrogen atom hydrogen bonding in a bifurcated manner to a pair of adjacent hydrogen-bond donors on the edge of the AT base pairs [3]. Most studies focused on the development of DNA-binding ligands with high affinity and sequence specificity. Among them, a series of head-to-head linked bis-benzimidazoles was reported as new sequence-selective DNA-binding agents [4]. Preliminary pharmacologic tests showed that these symmetrical bis-benzimidazoles were cytotoxic at the μM level, with activity significantly greater than that shown by Hoechst 33258 in a group of ovarian carcinoma cell lines. 

Most derivatives of Hoechst 33258 contained a phenyl group in 2-position of benzimidazole, which kept the whole molecule in a planar structure. No literature related to the use of derivatives of Hoechst 33258 with alkyl chain substituents at the 2-position of the benzimidazole has been reported so far. Recently, a series of symmetrical bis-benzimidazoles was designed by our group as novel MGBs, which linked pyridyl-methylenethio groups to the 2-position of bis-benzimidazole (submitted for publication). Antiproliferative evalutation in tumor cell lines showed that 2,2’-di-[[(3,5-dimethyl-4-methoxy)pyrid-2-yl]methylenethio]-5,5’-bis-1*H*,1’*H*-benzimidazole (**2**, Figure 1) effectively inhibited SKOV-3 cell proliferation with an IC_50_ value of 2.95 μM, which is more effective than that of Hoechst 33258. As a part of our ongoing program to design novel MGBs, herein, the synthesis and bioevaluation of dimeric proton pump inhibitor (PPI) analogues is reported. A molecular modeling study was performed to confirm the sequence selectivity of these compounds to AT base pairs in DNA.

## Results and Discussion

### Synthesis

**Scheme 1:**
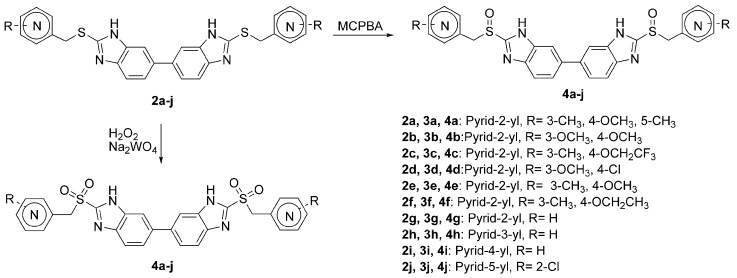
Synthesis of the presented compounds.

The synthetic route was as shown in Scheme 1. Reaction of the appropriate thio-ethers **2a–j** with *m*‑chloroperbenzoic acid (MCPBA) led to compounds **3a–j** in moderate yields. When this oxidant was replaced by hydrogen peroxide and the reaction was catalyzed by sodium tungstate, compounds **4a–j** were obtained in moderate to high yields. The synthesis of **2a–j** has been reported before [5]. 

### Biological evaluation

Bioactivity of these compounds on HeLa and BGC-823 cell proliferation was investigated *in vitro*. As shown in Table 1, for the HeLa cell line, compounds **3c**, **3h** and **3j** inhibited cell proliferation below a concentration of 10 μM, which makes them equally effective as cisplatin. Compounds **3a**, **3b**, **3d**, **3g**, **4a**, and **4d** were less potent, with inhibitory concentrations below 20 μM. Compounds **3f**, **4b**, and **4j** were much less potent, with inhibitory concentrations below 100 μM. The other eight compounds and compound **2** showed little effects under the same conditions. For the BGC-823 cell line, compounds **3d**, **3h**, and **3j** were as effective as cisplatin, and the inhibitory concentrations of **3a**, **3i**, **4d** and **4j** were under 100 μM. However, all the other thirteen compounds and compound **2** were much less effective. 

For compounds **2**, **3a**, and **4a**, the only difference was the oxidative state of sulfur atom. The anti-tumor test clearly showed that the sulfoxide was more effective than the corresponding sulfone and sulfide. This may be due to the fact that the sulfinyl compound **3a** has a similar sulfinyl structure as omeprazole and the latter could inhibit the V-H^+^-ATPase of tumor cells with a sulfoxide present. As a result of the inhibition, the PPI pretreatment would sensitize tumor cell lines to the effects of anti-tumor agents [6], therefore, the sulfoxide may show increased activity by inhibiting the V-H^+^-ATPase of tumor cells. Compounds **3g**, **3h** and **3i** share almost identical structures, except for the position of the pyridyl group nitrogen. Pharmacological results demonstrated that the pyrid-2-yl compound showed lower activity than the pyrid-3-yl and pyrid-4-yl compounds. This may be explained by the fact that that the nitrogen atom in the pyrid-2-yl derivative could form an intramolecular hydrogen-bond with the benzimidazole proton. Such hydrogen bonding would make the crucial benzimidazole protons less available for hydrogen bonding with DNA bases [7] and reduce the binding affinity of the pyrid-2-yl compound. Substituents on the pyridyl group were also important for the antiproliferative activity in pyrid-2-yl compounds. Substituted pyrid-2-yl compounds were more effective than non-substituted ones. The presence of substituents may increase the binding affinity by changing the conformation of compounds and interfering with the formation of the intramolecular hydrogen bond. As is known, halogen substituents may improve the biological activity of compounds. In the pharmacological test, compounds **3c**, **3d**, **3j**, **4d** and **4j** containing halogen-substituents in the pyridyl group exhibited more effective activity. 

**Table 1:** Structure and anti-tumor activity of dimeric PPI analogues against HeLa and BGC-823 tumor cell lines.

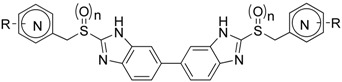						
Compd.	Substitutents				IC_50 _(μM)	
	Pyridyl	R		n	HeLa	BGC-823
**cisplatin**					1.6	1.3
**2**	Pyrid-2-yl		3-CH_3_, 4-OCH_3_, 5-CH_3_	0	> 100	> 100
**3a**	Pyrid-2-yl		3-CH_3_, 4-OCH_3_, 5-CH_3_	1	10.4	56.5
**3b**	Pyrid-2-yl		3-OCH_3_, 4-OCH_3_	1	12.8	> 100
**3c**	Pyrid-2-yl		3-CH_3_, 4-OCH_2_CF_3_	1	2.3	> 100
**3d**	Pyrid-2-yl		3-OCH_3_, 4-Cl	1	13.5	4.1
**3e**	Pyrid-2-yl		3-CH_3_, 4-OCH_3_	1	>100	> 100
**3f**	Pyrid-2-yl		3-CH_3_, 4-OCH_2_CH_3_	1	85.0	> 100
**3g**	Pyrid-2-yl		H	1	>100	> 100
**3h**	Pyrid-3-yl		H	1	4.9	5.6
**3i**	Pyrid-4-yl		H	1	14.9	30.1
**3j**	Pyrid-5-yl		2-Cl	1	5.5	0.8
**4a**	Pyrid-2-yl		3-CH_3_, 4-OCH_3_, 5-CH_3_	2	17.0	> 100
**4b**	Pyrid-2-yl		3-OCH_3_, 4-OCH_3_	2	30.9	> 100
**4c**	Pyrid-2-yl		3-CH_3_, 4-OCH_2_CF_3_	2	>100	> 100
**4d**	Pyrid-2-yl		3-OCH_3_, 4-Cl	2	10.2	67.9
**4e**	Pyrid-2-yl		3-CH_3_, 4-OCH_3_	2	>100	> 100
**4f**	Pyrid-2-yl		3-CH_3_, 4-OCH_2_CH_3_	2	>100	> 100
**4g**	Pyrid-2-yl		H	2	>100	> 100
**4h**	Pyrid-3-yl		H	2	>100	> 100
**4i**	Pyrid-4-yl		H	2	>100	> 100
**4j**	Pyrid-5-yl		2-Cl	2	34.5	56.7

### Molecular modeling

**Figure 2:**
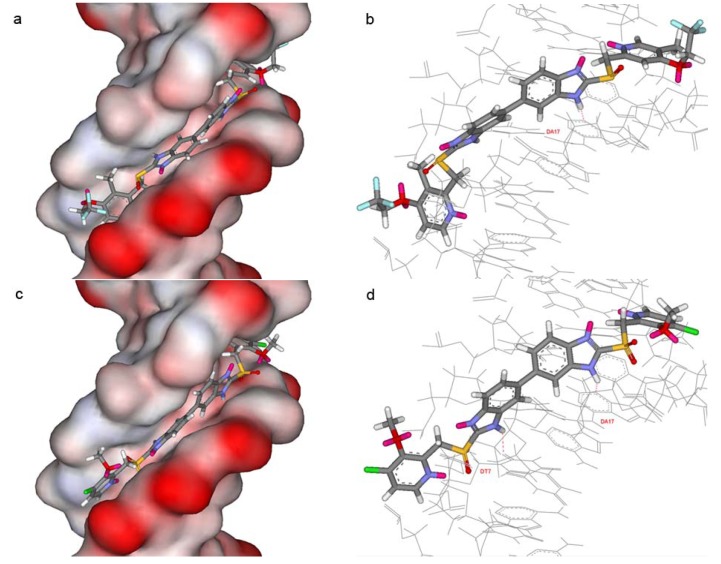
Two views of the binding to the sequence d(CGCAAATTTGCG) of compounds **3c** and **4d**, respectively. (a) Close-up view of compound **3c** binding in the minor groove, highlighting the electrostatic potential surface of the oligonucleotide. (b) Close-up view of hydrogen bonds between compound **3c** and the DNA minor groove. (c) Close-up view of compound **4d** binding in the minor groove, highlighting the electrostatic potential surface of the oligonucleotide. (d) Close-up view of hydrogen bonds between compound **4d** and the DNA minor groove.

A molecular docking study was performed in order to confirm the sequence selectivity of the dimeric PPI analogues. The representative compounds **3c** and **4d** were selected for docking with the minor groove of DNA. These molecular modeling studies were carried using the Sybyl/FlexX program. Usually electrostatic, van der Waals, hydrophobic and hydrogen bonding forces dominate the binding ability of small molecules, whereas sequence specificity is often attributed to the formation of key hydrogen bonds between a base pair and the small molecule. Another crucial structural requirement is that the ligand have a crescent shape and be able to adopt an “isohelical” conformation to fit the minor groove [3]. The X-ray crystallographic structure of the DNA dodecamer d(CGCAAATTTGCG) with a bifurcated hydrogen-bonded conformation of the AT base pairs and its complex with distamycin A was selected from the Protein Data Bank (PDB code: 2DND) for the docking study [8]. Two representative models of the dodecamer complexed to compounds **3c** and **4d** are shown in Figure 2. 

There were van der Waals contacts between compounds **3c**, **4d** and the narrow minor groove, respectively. Compounds **3c** and **4d** adopted concave shapes, which fitted exactly into the convex minor groove in the models. These two factors indicated that compounds **3c** and **4d** were able to penetrate deeply into the minor groove of DNA. In addition, hydrogen bonds between compounds **3c**, **4d** and DNA were formed, respectively. For compound **3c**, one hydrogen bond was formed between one benzimidazole NH group and T17-N3 (1.53 Å). For compound **4d**, two hydrogen bonds were formed. One was between the benzimidazole NH group and A17-N3 (1.69 Å), and the other was between the benzimidazole NH group and T7-O2 (2.40 Å). Therefore, compounds **3c** and **4d** can effectively and selectively bind to the central AT region in the minor groove. Some other compounds with poor antiproliferative effects were also docked into the DNA minor groove. In these models, the binding affinities of most compounds were relatively lower than those of compounds **3c** and **4d**. This means that the antiproliferative activity of these compounds *in vitro* may be related to their abilities to specifically binding at AT sites in DNA sequence.

## Conclusions

In summary, dimeric PPI analogues were synthesized as a novel type of DNA minor groove binders. Antiproliferative activities against HeLa and BGC-823 cell lines indicated that most of these compounds were effective, and some compounds showed μM level activity. Molecular docking was used to model the examine the binding of compounds **3c** and **4d** with the DNA minor groove. The results showed that these compounds could effectively fit into the minor groove and selectively bond with AT base pairs. Further studies in this area are in progress and will be reported upon in the future.

## Experimental Section

### General

All commercially available reagents and solvents were used without further purification unless otherwise specified. Solvents were dried and re-distilled prior to use according to standard methods. Melting points were determined on a Büchi Melting Point B-540 apparatus (Büchi Labortechnik, Flawil, Switzerland) and are uncorrected. ^1^H-NMR spectra were measured in DMSO-*d*_6_ on a Bruker ARX 300 spectrometer (Bruker, Rheinstetten, Germany). Chemical shifts are reported in parts per million (ppm) using tetramethylsilane (TMS) as internal standard if not specifically mentioned (*J* in Hz). Mass spectra were obtained on Waters Micromass^®^ Quattro Micro^TM^ API mass spectrometer (Waters Corporation, Milford, United States). Column chromatography was performed on silica gel H and analytical TLC on silica gel HF254 plates.

### 2,2’-di-[[(3,5-Dimethyl-4-methoxy)pyrid-2-yl]methylenesulfinyl]-5,5’-bis-1H,1’H-benzimidazole (**3a**).

Compound **2a** (1.0 g, 1.68 mmol) in dichloromethane (30 mL) was stirred in an ice-salt bath. When the temperature reached -15°C, MCPBA (0.71 g, 3.50 mmol) was added in batches. Afterwards the mixture was stirred for 1 hr. Saturated sodium carbonate solution (10 mL) was added to adjust the pH to about 9. The organic layer was separated and the aqueous was extracted with dichloromethane (20 mL × 3). The organic phases were combined and dried over anhydrous sodium sulfate. An oil was obtained after concentration, which was then purified by silica gel column chromatography (eluent CH_2_Cl_2_-CH_3_OH= 60:1) to give compound **3a**. Yield: 72 %; mp. = 158°C dec; ^1^H-NMR: δ = 13.66 (s, 1H, Bz-N*H*), 8.20 (s, 1H, Py-6-*H*), 7.67–8.01 (m, 3H, Bz-4,6,7-*H*), 4.78 (dd, 2H, *J*=19.8/13.8, SOC*H*_2_), 3.70 (s, 3H, OC*H*_3_), 2.20 (s, 6H, Py-3,5-C*H*_3_); MS (ESI-): m/e = 629.5 [M + 1]. 

Compounds **3b – 3j** were similarly prepared:

*2,2’-di-[[(3,4-Dimethoxy)pyrid-2-yl]methylenesulfinyl]-5,5’-bis-1H,1’H-benzimidazole* (**3b**). Yield: 70 %; mp. = 127 – 130°C; ^1^H-NMR: δ = 13.68 (s, 1H, Bz-N*H*), 8.16 (d, 1H, *J*=5.5, Py-6-*H*), 7.67–8.00 (m, 3H, Bz-4,6,7-*H*), 7.11 (d, 1H, *J*=5.6, Py-5-*H*), 4.72 (dd, 2H, *J*=18.1/13.0, SOC*H*_2_), 3.89 (s, 3H, OC*H*_3_), 3.78 (s, 3H, OC*H*_3_); MS (ESI-): m/e = 633.2 [M + 1].

*2,2’-di-[[[3-Methyl-4-(2,2,2-trifluoroethoxy)]pyrid-2-yl]methylenesulfinyl]-5,5’-bis-1H,1’H-benz-imidazole* (**3c**). Yield: 77 %; mp. = 172°C dec; ^1^H-NMR: δ = 13.68 (s, 1H, Bz-N*H*), 8.31 (d, 1H, J=5.7, Py-6-*H*), 7.88 (s, 1H, Bz-4-*H*), 7.73 (d, 1H, *J*=8.7, Bz-7-*H*), 7.62 (d, 1H, *J*=8.7, Bz-6-*H*), 7.10 (d, 1H, *J*=5.7, Py-5-*H*), 4.93 (q, 2H, *J*=8.7, CF_3_C*H*_2_O), 4.81 (dd, 2H, *J*=33.5/13.7, SOC*H*_2_), 2.21 (s, 3H, Py-3-C*H*_3_); MS (ESI-): m/e = 771.5 [M + 35].

*2,2’-di-[[(3-Methoxy-4-chloro)pyrid-2-yl]methylenesulfinyl]-5,5’-bis-1H,1’H-benzimidazole* (**3d**). Yield: 68 %; mp. = 130–133°C; ^1^H-NMR: δ = 13.72 (s, 1H, Bz-N*H*), 8.29 (d, 1H, *J*=5.1, Py-6-*H*), 7.62 – 7.78 (m, 3H, Bz-4,6,7-*H*), 7.61 (d, 1H, *J*=5.1, Py-5-*H*), 4.85 (s, 2H, SOC*H*_2_), 3.90 (s, 3H, OC*H*_3_); MS (ESI-): m/e = 639.2 [M – 1].

*2,2’-di-[[(3-Methyl-4-methoxy)pyrid-2-yl]methylenesulfinyl]-5,5’-bis-1H,1’H-benzimidazole* (**3e**). Yield: 78 %; mp. = 203°C dec; ^1^H-NMR: δ = 13.61 (s, 1H, Bz-N*H*), 8.26 (d, 1H, *J*=1.5, Py-6-*H*), 7.60 –8.10 (m, 3H, Bz-4,6,7-*H*), 6.98 (d, 1H, *J*=1.5, Py-5-*H*), 4.83 (dd, 2H, *J*=22.5/9.3, SOC*H*_2_), 3.87 (s, 3H, OC*H*_3_), 2.16 (s, 3H, Py-3-C*H*_3_); MS (ESI-): m/e = 599.4 [M – 1].

*2,2’-di-[[(3-Methyl-4-ethoxy)pyrid-2-yl]methylenesulfinyl]-5,5’-bis-1H,1’H-benzimidazole* (**3f**). Yield: 71 %; mp. = 95–98°C; ^1^H-NMR: δ = 13.65 (s, 1H, Bz-N*H*), 8.22 (d, 1H, *J*=5.4, Py-6-*H*), 7.60–7.80 (m, 3H, Bz-4,6,7-*H*), 6.95 (d, 1H, *J*=5.4, Py-5-*H*), 4.78 (dd, 2H, *J*=24.6/13.8, SOC*H*_2_), 4.12 (q, 2H, *J*=6.9, CH_3_C*H*_2_O), 2.15 (s, 3H, Py-3-C*H*_3_), 1.35 (t, 3H, *J*=6.9, C*H*_3_CH_2_O); MS (ESI-): m/e = 627.5 [M – 1].

*2,2’-di-[(Pyrid-2-yl)methylenesulfinyl]-5,5’-bis-1H,1’H-benzimidazole* (**3g**). Yield: 81 %; mp. = 198°C dec; ^1^H-NMR: δ = 13.63 (s, 1H, Bz-N*H*), 8.54 (d, 1H, *J*=4.2, Py-6-*H*), 7.60–7.85 (m, 4H, Bz-4,6,7-*H*, Py-4-*H*), 7.30–7.40 (m, 2H, Py-3,5-*H*), 4.77 (dd, 2H, *J*=26.4/12.9, SOC*H*_2_); MS (ESI-): m/e = 511.2 [M – 1].

*2,2’-di-[(Pyrid-3-yl)methylenesulfinyl]-5,5’-bis-1H,1’H-benzimidazole* (**3h**). Yield: 76 %; mp. = 211°C dec; ^1^H-NMR: δ = 13.42 (s, 1H, Bz-N*H*), 8.47 (d, 1H, *J*=3.6, Py-6-*H*), 8.26 (s, 1H, Py-2-*H*), 7.58–8.08 (m, 3H, Bz-4,6,7-*H*), 7.52 (d, 1H, *J*=7.8, Py-4-*H*), 7.30 (dd, 1H, *J*=7.8/4.8, Py-5-*H*), 4.68 (dd, 2H, J=71.4/13.2, SOC*H*_2_); MS (ESI-): m/e = 513.1 [M + 1].

*2,2’-di-[(Pyrid-4-yl)methylenesulfinyl]-5,5’-bis-1H,1’H-benzimidazole* (**3i**). Yield: 83 %; mp. = 172°C dec; ^1^H-NMR: δ = 13.48 (s, 1H, Bz-N*H*), 8.46 (d, 2H, *J*=4.8, Py-2,6-*H*), 7.60 – 8.10 (m, 3H, Bz-4,6,7-*H*), 7.14 (d, 2H, *J*=5.1, Py-3,5-*H*), 4.67 (dd, 2H, *J*=68.7/12.9, SOC*H*_2_); MS (ESI-): m/e = 511.3 [M – 1].

*2,2’-di-[[(2-Chloro)pyrid-5-yl]methylenesulfinyl]-5,5’-bis-1H,1’H-benzimidazole* (**3j**). Yield: 64 %; mp. = 166 – 169°C; ^1^H-NMR: δ = 13.50 (s, 1H, Bz-N*H*), 8.06 (d, 1H, *J*=2.0, Py-6-*H*), 7.60–8.00 (m, 3H, Bz-4,6,7-*H*), 7.53 (dd, 1H, *J*=8.3/2.2, Py-4-*H*), 7.45 (d, 1H, *J*=8.2, Py-3-*H*), 4.70 (dd, 2H, *J*=77.6/13.2, SOC*H*_2_); MS (ESI-): m/e = 579.3 [M – 1].

### 2,2’-di-[[(3,5-dimethyl-4-methoxy)pyrid-2-yl]methylenesulfonyl]-5,5’-bis-1H,1’H-benzimidazole (**4a**).

To a solution of **2a** (1.0 g, 1.70 mmol) in THF (30 mL), sodium tungstate (0.15 g, 0.45 mmol), distilled water (10 mL) and hydrogen peroxide (30 %, 5 mL, 44.1 mmol) were added. The mixture was stirred at 50°C for 5 hrs. After the solvent was evaporated under vacuum, water (50 mL) was added to the resultant oil. The white solid obtained after filtration was dried. Purification by gel column chromatography (eluent CH_2_Cl_2_-CH_3_OH= 80:1) gave compound **4a**. Yield: 82 %; mp. =167 – 170°C; ^1^H-NMR: δ = 13.89 (s, 1H, Bz-N*H*), 8.05 (s, 1H, Py-6-*H*), 7.79 – 8.04 (m, 3H, Bz-4,6,7-*H*), 5.11 (s, 2H, SO_2_C*H*_2_), 3.71 (s, 3H, OC*H*_3_), 2.26 (s, 3H, Py-3-C*H*_3_), 2.19 (s, 3H, Py-5-C*H*_3_); MS (ESI-): m/e = 661.5 [M + 1].

Compounds **4b – 4j** were similarly prepared:

*2,2’-di-[[(3,4-Dimethoxy)pyrid-2-yl]methylenesulfonyl]-5,5’-bis-1H,1’H-benzimidazole* (**4b**). Yield: 85 %; mp. = 173 – 175°C; ^1^H-NMR: δ = 13.88 (s, 1H, Bz-N*H*), 7.99 (d, 1H, *J*=5.5, Py-6-*H*), 7.77–8.01 (m, 3H, Bz-4,6,7-*H*), 7.09 (d, 1H, *J*=5.5, Py-5-*H*), 5.01 (s, 2H, SO_2_C*H*_2_), 3.88(s, 3H, OC*H*_3_), 3.78 (s, 3H, OC*H*_3_); MS (ESI-): m/e = 665.5 [M + 1].

*2,2’-di-[[[3-Methyl-4-(2,2,2-trifluoroethoxy)]pyrid-2-yl]methylenesulfonyl]-5,5’-bis-1H,1’H-benz-imidazole* (**4c**). Yield: 88 %; mp. = 259–262°C; ^1^H-NMR: δ = 13.88 (s, 1H, Bz-N*H*), 8.13 (d, 1H, *J*=5.7, Py-6-*H*), 7.76–8.00 (m, 3H, Bz-4,6,7-*H*), 7.08 (d, 1H, *J*=5.7, Py-5-*H*), 5.15 (s, 2H, SO_2_C*H*_2_), 4.91 (q, 2H, *J*=8.7, CF_3_C*H*_2_O), 2.25 (s, 3H, Py-3-C*H*_3_); MS (ESI-): m/e = 769.5 [M + 1].

*2,2’-di-[[(3-Methoxy-4-chloro)pyrid-2-yl]methylenesulfonyl]-5,5’-bis-1H,1’H-benzimidazole* (**4d**). Yield: 73 %; mp. = 160–163°C; ^1^H-NMR: δ = 13.93 (s, 1H, Bz-N*H*), 8.12 (d, 1H, *J*=5.1, Py-6-*H*), 7.60–8.00 (m, 3H, Bz-4,6,7-*H*), 7.61 (d, 1H, *J*=5.1, Py-5-*H*), 5.14 (s, 2H, SO_2_C*H*_2_), 3.91 (s, 3H, OC*H*_3_); MS (ESI-): m/e = 671.4 [M – 1].

*2,2’-di-[[(3-Methyl-4-methoxy)pyrid-2-yl]methylenesulfonyl]-5,5’-bis-1H,1’H-benzimidazole* (**4e**). Yield: 81 %; mp. = 240 – 243°C; ^1^H-NMR: δ = 13.89 (s, 1H, Bz-N*H*), 8.26 (d, 1H, *J*=5.7, Py-6-*H*), 7.35–7.80 (m, 3H, Bz-4,6,7-*H*), 6.99 (d, 1H, *J*=5.7, Py-5-*H*), 4.94 (s, 2H, SO_2_C*H*_2_), 3.86 (s, 3H, OC*H*_3_), 2.16 (s, 3H, Py-3-C*H*_3_); MS (ESI-): m/e = 633.4 [M + 1].

*2,2’-di-[[(3-Methyl-4-ethoxy)pyrid-2-yl]methylenesulfonyl]-5,5’-bis-1H,1’H-benzimidazole* (**4f**). Yield: 82%; mp. = 179 – 182°C; ^1^H-NMR: δ = 13.86 (s, 1H, Bz-N*H*), 8.05 (d, 1H, *J*=5.7, Py-6-*H*), 7.70–8.00 (m, 3H, Bz-4,6,7-*H*), 6.94 (d, 1H, *J*=5.7, Py-5-*H*), 5.10 (s, 2H, SO_2_C*H*_2_), 4.12(q, 2H, *J*=6.9, CH_3_C*H*_2_O), 2.21 (s, 3H, Py-3-C*H*_3_), 1.36 (t, 3H, *J*=6.9, C*H*_3_CH_2_O); MS (ESI-): m/e = 661.4 [M + 1].

*2,2’-di-[(Pyrid-2-yl)methylenesulfonyl]-5,5’-bis-1H,1’H-benzimidazole* (**4g**). Yield: 86 %; mp. = 203–206°C; ^1^H-NMR: δ = 13.90 (s, 1H, Bz-N*H*), 8.41 (d, 1H, *J*=4.5, Py-6-*H*), 7.75–8.00 (m, 4H, Bz-4,6,7-*H*, Py-4-*H*), 7.42 (d, 1H, *J*=7.8, Py-3-*H*), 7.34 (dd, 1H, *J*=4.5/4.5, Py-5-*H*), 5.11 (s, 2H, SO_2_C*H*_2_); MS (ESI-): m/e = 543.4 [M – 1].

*2,2’-di-[(Pyrid-3-yl)methylenesulfonyl]-5,5’-bis-1H,1’H-benzimidazole* (**4h**). Yield: 80 %; mp. = 254– 258°C; ^1^H-NMR: δ = 13.91 (s, 1H, Bz-N*H*), 8.49 (d, 1H, *J*=4.6, Py-6-*H*), 8.42 (s, 1H, Py-2-*H*), 7.60–7.83 (m, 3H, Bz-4,6,7-*H*), 7.55 (d, 1H, *J*=8.5, Py-4-*H*), 7.30–7.50 (m, 1H, Py-5-*H*), 4.95 (s, 2H, SO_2_C*H*_2_); MS (ESI-): m/e = 543.4 [M – 1].

*2,2’-di-[(Pyrid-4-yl)methylenesulfonyl]-5,5’-bis-1H,1’H-benzimidazole* (**4i**). Yield: 81 %; mp. = 231– 234°C; ^1^H-NMR: δ = 13.90 (s, 1H, Bz-N*H*), 8.52 (d, 2H, *J*=4.5, Py-2,6-*H*), 7.70–8.10 (m, 3H, Bz-4,6,7-*H*), 7.25 (d, 2H, *J*=4.6, Py-3,5-*H*), 5.11 (s, 2H, SO_2_C*H*_2_); MS (ESI-): m/e = 543.4 [M – 1].

*2,2’-di-[[(2-Chloro)pyrid-5-yl]methylenesulfonyl]-5,5’-bis-1H,1’H-benzimidazole* (**4j**). Yield: 77 %; mp. = 241–244°C; ^1^H-NMR: δ = 14.01 (s, 1H, Bz-N*H*), 8.24 (d, 1H, *J*=1.5, Py-6-*H*), 7.70–8.20 (m, 3H, Bz-4,6,7-*H*), 7.74 (dd, 1H, *J*=8.3/2.0, Py-4-*H*), 7.54 (d, 1H, *J*=8.3, Py-3-*H*), 5.16 (s, 2H, SO_2_C*H*_2_); MS (ESI-): m/e = 611.4 [M – 1].

### Molecular modeling

The molecular modeling studies were performed with the Sybyl 6.9.1 package (Tripos Inc., St. Louis, MO) on a SGI Fuel Workstation with the Irix 6.5 platform. All molecular structures were constructed and geometry optimized *in vacuo* (ε = 1) with the implemented Tripos force field using the Powell method until the convergence criterion of 0.05 kcal/mol change in energy between successive iterations was reached. The charges were calculated by the Gasteiger-Hückel method. Docking studies were performed with FlexX 1.6. During the docking process, the binding site atoms and the ligand atoms were set to be flexible. An incremental construction algorithm was applied to generate and minimize the possible pose. The models were visualized with DS Visualizer v1.5 (Accelrys Inc., San Diego, CA).

### Biological assays

The anti-proliferational effects of HeLa cells and BGC-823 cells were tested by the same methods. Tumor cells in RPMI1640 medium with 10 % fetal bovine serum were plated in 96-well microtiter plates (4.0 × 104 cells per well), and allowed to adhere at 37°C with 5 % CO_2_ for 4 h. The test compound was then added, and the cells were incubated at 37°C with 5 % CO_2_ for 72 h. The cell viability was assessed using a standard MTT assay [9].
